# Inhibition of Autophagy at Different Stages by ATG5 Knockdown and Chloroquine Supplementation Enhances Consistent Human Disc Cellular Apoptosis and Senescence Induction rather than Extracellular Matrix Catabolism

**DOI:** 10.3390/ijms22083965

**Published:** 2021-04-12

**Authors:** Masaaki Ito, Takashi Yurube, Yutaro Kanda, Yuji Kakiuchi, Yoshiki Takeoka, Toru Takada, Ryosuke Kuroda, Kenichiro Kakutani

**Affiliations:** 1 Department of Orthopaedic Surgery, Kobe University Graduate School of Medicine, 7-5-1 Kusunoki-cho, Chuo-ku, Kobe 650-0017, Japan; maito28710@yahoo.co.jp (M.I.); youthfuldays_y_k@yahoo.co.jp (Y.K.); yuji_uz_7@yahoo.co.jp (Y.K.); yoshiki_tkk@hotmail.com (Y.T.); kurodar@med.kobe-u.ac.jp (R.K.); kakutani@med.kobe-u.ac.jp (K.K.); 2 Department of Orthopaedic Surgery, Kobe Hokuto Hospital, 37-3 Yamada-cho Shimotanigami Aza Umekidani, Kita-ku, Kobe 651-1243, Japan; takada-t@hokuto-hp.or.jp

**Keywords:** intervertebral disc nucleus pulposus cells, autophagy, autophagy-related gene 5 (ATG5), chloroquine, RNA interference (RNAi), spine

## Abstract

The intervertebral disc is the largest avascular organ. Autophagy is an important cell survival mechanism by self-digestion and recycling damaged components under stress, primarily nutrient deprivation. Resident cells would utilize autophagy to cope with the harsh disc environment. Our objective was to elucidate the roles of human disc cellular autophagy. In human disc cells, serum deprivation and pro-inflammatory interleukin-1β (IL-1β) stimulation increased autophagy marker microtubule-associated protein 1 light chain 3 (LC3)-II and decreased autophagy substrate p62/sequestosome 1 (p62/SQSTM1), indicating enhanced autophagy. Then, RNA interference (RNAi) of autophagy-related gene 5 (ATG5), essential for autophagy, showed decreases in ATG5 protein (26.8%–27.4%, *p* < 0.0001), which suppressed early-stage autophagy with decreased LC3-II and increased p62/SQSTM1. Cell viability was maintained by ATG5 RNAi in serum-supplemented media (95.5%, *p* = 0.28) but reduced in serum-free media (80.4%, *p* = 0.0013) with IL-1β (69.9%, *p* = 0.0008). Moreover, ATG5 RNAi accelerated IL-1β-induced changes in apoptosis and senescence. Meanwhile, ATG5 RNAi unaffected IL-1β-induced catabolic matrix metalloproteinase release, down-regulated anabolic gene expression, and mitogen-activated protein kinase pathway activation. Lysosomotropic chloroquine supplementation presented late-stage autophagy inhibition with apoptosis and senescence induction, while catabolic enzyme production was modest. Disc-tissue analysis detected age-related changes in ATG5, LC3-II, and p62/SQSTM1. In summary, autophagy protects against human disc cellular apoptosis and senescence rather than extracellular matrix catabolism.

## 1. Introduction

Up to 85% of people experience back pain during their lives [1]. Back pain causes disability that increases medical expenses and affects the workforce [1]. Health care costs related to back pain are approximately $100 billion/year in the US [2]. Although back pain is multifactorial, intervertebral disc degeneration is an independent cause [3]. Currently, surgical resection is the primary treatment for degenerative discs, resulting in the loss of shock absorption and spinal movement [4]. Therefore, the development of new therapeutic strategies to prevent disc degeneration is highly demanded.

The intervertebral disc consists of the nucleus pulposus (NP) encapsulated by the annulus fibrosus (AF) and endplates. The disc is anatomically the largest avascular organ in the body [4]; therefore, nutrient supply depends on the diffusion through the endplates. Endplate calcification and subchondral bone sclerosis with aging can limit nutrient supply [5]. Additional nutrient deprivation is a suspected contributor to disc degeneration [5].

Intervertebral disc degeneration is biochemically characterized by extracellular matrix degradation [4,6]. Matrix metabolism is regulated by the balance between catabolic enzymes, primarily matrix metalloproteinases (MMPs), and their anti-catabolic inhibitors, tissue inhibitors of metalloproteinases (TIMPs) [7]. Increased MMPs relative to TIMPs are often observed in human clinical [7] and rodent experimental disc degeneration [8,9,10], leading to degraded matrix components including proteoglycans, principally aggrecan, and collagens, predominantly types II in the NP and I in the AF [6].

Another hallmark of disc degeneration is decreased cellularity, primarily resulting from programmed cell death, apoptosis [11]. A high apoptosis incidence has been observed in human [12] and rodent disc aging and degeneration [10,13]. Findings of irreversible cell growth arrest by aging, senescence [14], also increase with human disc degeneration [15]. Furthermore, autophagy, the intracellular process by which cells break down and recycle damaged components [16,17], has increased attention in the disc [18]. Autophagy is an important cell survival mechanism to sustain metabolism and prevent the accumulation of damaged toxic proteins and organelles under stress, including nutrient deprivation [16,17]. We thus hypothesized that resident cells would utilize autophagy to cope with the harsh, low-nutrient disc environment [5].

**Figure 1:**
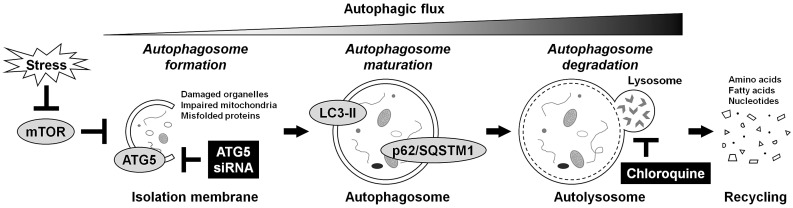
Schematic illustration of autophagy regulation. Autophagy is an intracellular mechanism by self-digestion and recycling damaged components in response to various stresses, including nutrient deprivation. Under stress conditions, mTOR is suppressed, and autophagy-related proteins are activated for the formation, growth, and closure of the isolation membrane, in which ATG5 is essential. The ATG5 is required for the lipidation of LC3 from LC3-I to LC3-II. The LC3-II is the only protein that exists on the completed double-membraned isolation membrane, autophagosome. Then, matured autophagosomes that incorporate their own surplus protein and waste products are degraded by the fusion with the lysosome, leading to the production and reuse of amino acids. The p62/SQSTM1 serves as a link with LC3, and its labeled proteins are selectively degraded in this process. Monitoring this dynamic process, autophagic flux, is essential for understanding autophagy regulation. In this study, autophagy was inhibited at the early stage by RNAi using ATG5 siRNA or at the late stage by lysosomotropic chloroquine supplementation.

Under physiological conditions, basal autophagy acts in the intracellular self-renovating quality control [17]. Under stress conditions, autophagy-related genes (ATGs) are activated for autophagosome formation and maturation [16]. The nucleation, elongation, and closure of the isolation membrane occur to form the double-membraned autophagosome. The autophagosome then fuses with the lysosome, called the autolysosome, upon which the enclosed cargo is degraded, and its constituents are released and reutilized as induced autophagy. The microtubule-associated protein 1 light chain 3 (LC3) (mammalian Atg8 homolog) is a ubiquitin-like protein, present in the cytosolic form, LC3-I, or phosphatidylethanolamine-conjugated form, LC3-II [19]. The LC3-II is the only protein marker reliably associated with autophagosome maturation. p62/sequestosome 1 (p62/SQSTM1) is a ubiquitin-binding protein serving as a link between LC3 and ubiquitinated substrates [19]. The p62/SQSTM1 and p62/SQSTM1-bound polyubiquitinated proteins become incorporated into the completed autophagosome and are degraded in the autolysosome, thus demonstrating an inverse correlation with autophagosome degradation levels. Furthermore, ATG5 is a key protein involved in isolation membrane expansion, essential for autophagosome formation [19]. The ATG5 is activated by ATG7 and forms the conjugation with ATG12. The ATG12–ATG5 conjugation acts as an E3-like enzyme required for LC3 lipidation. Monitoring of this dynamic process, autophagic flux [19], is critical for understanding the roles of autophagy (Figure 1).

In molecular signaling, autophagy has the negative control of the mammalian target of rapamycin (mTOR) [20]. The mTOR is a serine/threonine kinase that integrates nutrients to execute cell growth and division [20]. Down-stream effectors of mTOR complex 1 (mTORC1), including p70/ribosomal S6 kinase (p70/S6K), regulate cell proliferation, messenger RNA (mRNA) translation, and protein synthesis [20]. Then, mTORC1 directly engages in upstream regulation of Akt [20], essential for pro-survival by suppressing apoptosis [21]. The Akt phosphorylation is associated with the class-I phosphatidylinositol 3-kinase (PI3K), which promotes cell survival through the negative feedback loop from p70/S6K under mTORC1 suppression [22]. Recent studies have reported that mTORC1-inhibiting rapamycin extends mammalian lifespan [23]. Rapamycin has protective roles in human disc cells [24,25] and chondrocytes [26]. Cartilage-specific mTOR deletion enhances autophagy and protects against destabilized medial meniscus-induced osteoarthritis in mice [27]. Our human disc-cell study through post-transcriptional gene silencing by RNA interference (RNAi) has found protective effects of specific mTORC1 suppression against apoptosis, senescence, and matrix catabolism with increased Akt phosphorylation and enhanced autophagy [24]. Dual mTORC1 and Akt inhibition further identified these mTORC1 suppression-mediated cellular protection as Akt-dependent [25]. Based on the importance of mTOR as the central signal integrator for nutrition, extensive suppression would be harmful [20]. Homogeneous mTOR deletion reaches embryonic lethality [28]. Therefore, specific identification of mTOR-signaling cascade(s) exerting beneficial effects on target cells is necessary.

Unlike Akt, it is still unknown whether autophagy is truly protective toward disc cells. In prior studies, cell-protective contributions of autophagy have largely included confounders of mTOR-signaling modulation [26]. We thus designed an in vitro study to elucidate the roles of human disc cellular autophagy. However, uncontaminated loss-of-function of autophagy through pharmacological approaches is difficult. Although 3-methyadenine is often used to block autophagy through the class-III PI3K [19], it can promote autophagy through the class-I PI3K at suboptimal concentrations in long-term experiments [29]. Therefore, RNAi targeting ATG5 was applied to accomplish early-stage autophagy inhibition. Moreover, chloroquine was used for late-stage autophagy inhibition. Chloroquine raises the lysosomal/vacuolar pH, inhibiting the fusion between the autophagosome and lysosome into the autolysosome [19]. Gene-silencing and pharmacological approaches at different stages should clarify specific effects of autophagy on human disc cells. Additionally, the involvement of autophagy-related proteins in vivo was explored in human degenerative disc tissues, as autophagy levels based on aging remain unclear.

## 2. Results

### 2.1. Basal and Serum Deprivation and Inflammation-Induced Autophagy in Human Disc NP Cells

Human intervertebral disc NP cells were isolated from surgical specimens of patients with degenerative lumbar disease. To reduce possible variations based on degeneration grade, only Pfirrmann grade-3 and grade-4 discs were obtained [30]. Collected cells were cultured under 2% O_2_ to simulate the physiologically hypoxic disc environment [5]. To retain the phenotype, only first-passage cells are used in experiments [24,25,31].

**Figure 2:**
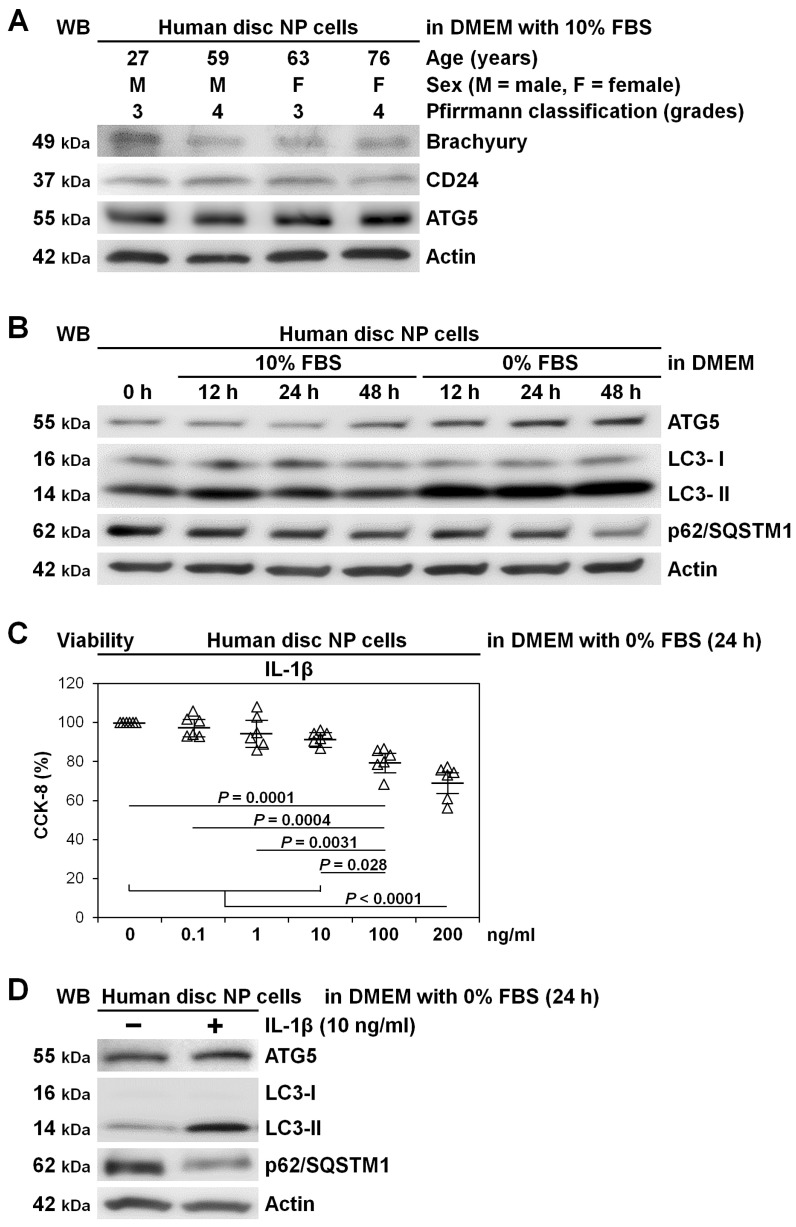
Basal and serum deprivation and inflammation-induced autophagy in human disc nucleus pulposus (NP) cells. (**A**) Western blotting for disc NP-phenotypic brachyury and CD24 and autophagic ATG5 in total protein extracts from human disc NP cells of patients who underwent lumbar spine surgery for degenerative disease after culturing for 240 h in Dulbecco’s modified Eagle’s medium (DMEM) with 10% FBS. Actin was used as a loading control. Immunoblots show samples randomly selected (*n* = 4). (**B**) Time-course Western blotting for autophagic ATG5, LC3, and p62/SQSTM1 and loading control actin in total protein extracts from human disc NP cells after up to 48 h culture in serum-supplemented DMEM with 10% FBS or serum-free DMEM with 0% FBS. Immunoblots shown are representative of experiments with similar results (*n* = 6). (**C**) Cell viability of human disc NP cells using CCK-8 after 24 h treatment of 0–200 ng/mL IL-1β in serum-free DMEM with 0% FBS. Changes in CCK-8 dehydrogenase activity of IL-1β treatment relative to the vehicle control are shown. Data are the mean ± 95% CI. One-way repeated-measures ANOVA and the Tukey–Kramer post-hoc test were used (*n* = 6). (**D**) Western blotting for autophagic ATG5, LC3, and p62/SQSTM1 and loading control actin in total protein extracts from human disc NP cells after 24 h culture in serum-free DMEM with or without 10 ng/mL IL-1β. Immunoblots shown are representative of experiments with similar results (*n* = 6).

First, to validate human disc NP cells, we assessed disc NP notochord-related marker expression. The notochordal origin of the disc NP results in relatively high specificity of brachyury and CD24 [32]. Western blotting demonstrated that human disc NP cells, at varying ages, consistently expressed brachyury, CD24, and also autophagy-essential ATG5 (Figure 2A).

Next, to confirm autophagy involvement in human disc NP cells, we assessed responses to serum starvation. Cells received media change to no fetal bovine serum (FBS)-containing Dulbecco’s modified Eagle’s medium (DMEM) [24,25,31] to mimic nutrient deprivation [5]. Time-course Western blotting showed persistently increased LC3-II and decreased p62/SQSTM1 in serum-free DMEM with 0% FBS, which is consistent with enhanced autophagy. In serum-supplemented DMEM with 10% FBS, LC3-II transiently increased but subsequently decreased, whereas decreases in p62/SQSTM1 were time-dependent, suggesting the involvement of self-renewal basal autophagy. Both conditions developed modest increases in ATG5 protein expression, corresponding to the severity of serum deprivation (Figure 2B).

We also assessed autophagy induction by pro-inflammatory interleukin-1 beta (IL-1β) stimulation in human disc NP cells. Cells additionally underwent IL-1β stimulation [24,25,26]. The IL-1β is a pro-inflammatory cytokine closely linked to the pathogenesis of disc degeneration [33], showing increased production with its severity [34]. Based on dose-dependent reductions in cell viability, 10 ng/mL IL-1β was selected as an effective but non-toxic concentration (Figure 2C). Supplemented IL-1β presented substantial autophagy induction with increased LC3-II, decreased p62/SQSTM1, and modestly increased ATG5 (Figure 2D).

### 2.2. Decreased Viability with Induced Apoptosis and Senescence by Autophagy Inhibition through ATG5 Knockdown in Human Disc NP Cells

**Figure 3:**
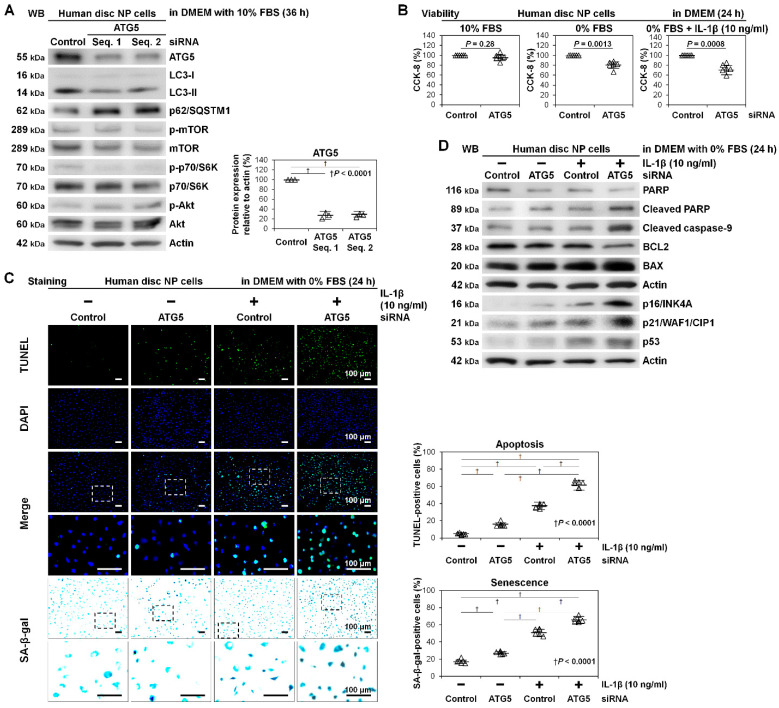
Decreased viability with induced apoptosis and senescence by autophagy inhibition through ATG5 knockdown in human disc NP cells. (**A**) Western blotting for autophagic ATG5, LC3, and p62/SQSTM1 and mTOR signaling-related mTOR, phosphorylated mTOR, p70/S6K, phosphorylated p70/S6K, Akt, and phosphorylated Akt in total protein extracts from human disc NP cells after a 36 h transfection of ATG5 siRNA with two different sequences or non-targeting siRNA in DMEM with 10% FBS. Actin was used as a loading control. Immunoblots shown are representative of experiments with similar results (*n* = 3). Changes in protein expression of ATG5 (normalized to actin) in ATG5 RNAi treatment relative to non-targeting siRNA-transfected control are shown. Data are the mean ± 95% CI. † *p* < 0.0001. One-way repeated-measures ANOVA and the Tukey-Kramer post-hoc test were used (*n* = 3). (**B**) Cell viability of human disc NP cells using CCK-8 after a 36 h transfection of ATG5 or non-targeting siRNA in DMEM with 10% FBS followed by 24 h culture in serum-supplemented DMEM with 10% FBS, serum-free DMEM with 0% FBS, or serum-free DMEM with 0% FBS and 10 ng/mL IL-1β. Changes in CCK-8 dehydrogenase activity of ATG5 RNAi treatment relative to non-targeting siRNA-transfected control are shown. Data are the mean ± 95% CI. A paired *t*-test was used (*n* = 6). (**C**) Fluorescence for apoptotic TUNEL (green), nuclear DAPI (blue), and merged signals and cytochemistry for senescent SA-β-gal in human disc NP cells after a 36 h transfection of ATG5 or non-targeting siRNA in 10% FBS-supplemented DMEM followed by culturing for 24 h in serum-free DMEM with or without 10 ng/mL IL-1β. The fluorescent and cytochemical images shown are representative of experiments with similar results (*n* = 6). Changes in the percentage of TUNEL-positive cells in DAPI-positive cells and of SA-β-gal-positive cells in total cells are shown. The number of cells was counted in six random low-power fields (×100). Data are the mean ± 95% CI. † *p* < 0.0001. One-way repeated-measures ANOVA and the Tukey–Kramer post-hoc test were used (*n* = 6). (**D**) Western blotting for pro-apoptotic cleaved PARP, cleaved caspase-9, and BAX, anti-apoptotic PARP and BCL2, pro-senescent p16/INK4A, p21/WAF1/CIP1, and p53, and loading control actin in total protein extracts from human disc NP cells after a 36 h transfection of ATG5 or non-targeting siRNA in 10% FBS-supplemented DMEM followed by 24 h culture in serum-free DMEM with or without 10 ng/mL IL-1β. Immunoblots shown are representative of experiments with similar results (*n* = 6).

After confirming the presence of basal and induced autophagy in human disc NP cells, we performed autophagy inhibition through RNAi of ATG5. To exclude off-target effects of RNAi, consistent findings were confirmed using at least two different small interfering RNA (siRNA) sequences in all following experiments. First, we examined knockdown efficacy of ATG5 RNAi, demonstrating reduced ATG5 protein expression—non-targeting siRNA, 100.0%; ATG5 siRNA no. 1, 26.8% ± 7.0% (*p* < 0.0001); ATG5 siRNA no. 2, 27.4% ± 4.2% (*p* < 0.0001). Both siRNAs against ATG5 were effective. Then, we assessed ATG5 RNAi-modified autophagy and mTOR signaling. In Western blotting, ATG5 RNAi decreased LC3-II and increased p62/SQSTM1 without changes in expression and phosphorylation of mTOR, p70/S6K, or Akt, indicating ATG5 knockdown-dependent early-stage autophagy (but not mTOR-signaling) inhibition (Figure 3A).

Next, we assessed whether ATG5 RNAi affected disc NP-cell viability. No significant differences in Cell Counting Kit-8 (CCK-8) dehydrogenase activity were detected between the control and ATG5 RNAi groups in 10% FBS-supplemented DMEM—non-targeting siRNA, 100.0%; ATG5 siRNA, 95.5 ± 5.6% (*p* = 0.28). However, there were significant differences in 0% FBS-supplemented DMEM—non-targeting siRNA, 100.0%; ATG5 siRNA, 80.4% ± 5.9% (*p* = 0.0013)—and additionally with IL-1β—non-targeting siRNA, 100.0%; ATG5 siRNA, 69.9% ± 7.8% (*p* = 0.0008) (Figure 3B).

Furthermore, we assessed whether ATG5 RNAi affected disc NP-cell death and aging. The percentage of apoptotic terminal deoxynucleotidyl transferase dUTP nick end labeling (TUNEL)-positive cells [35] increased by pro-inflammatory IL-1β stimulation (*p* < 0.0001), which was enhanced by ATG5 RNAi (*p* < 0.0001). Similarly, IL-1β-increased percentage of senescence-associated beta-galactosidase (SA-β-gal)-positive cells [36] (*p* < 0.0001) was amplified by ATG5 RNAi (*p* < 0.0001) (Figure 3C). In Western blotting, IL-1β-mediated inflammation modestly promoted poly (ADP-ribose) polymerase (PARP) [37] and caspase-9 [38] cleavage, increased B-cell lymphoma 2 (BCL2)-associated X protein (BAX) [39] expression, and decreased BCL2 [39] expression, indicating apoptosis induction through the mitochondrial pathway, and increased p16/INK4A [40], p21/WAF1/CIP1 [41], and p53 [42] expression, indicating senescence induction. The observed IL-1β-induced apoptosis and senescence were all exaggerated by ATG5 RNAi, supporting anti-apoptotic and anti-senescent roles of ATG5-dependent autophagy (Figure 3D).

### 2.3. Unaffected Matrix Catabolism and Mitogen-Activated Protein Kinase (MAPK)-Signaling Pathways by Autophagy Inhibition through ATG5 Knockdown in Human Disc NP Cells

**Figure 4:**
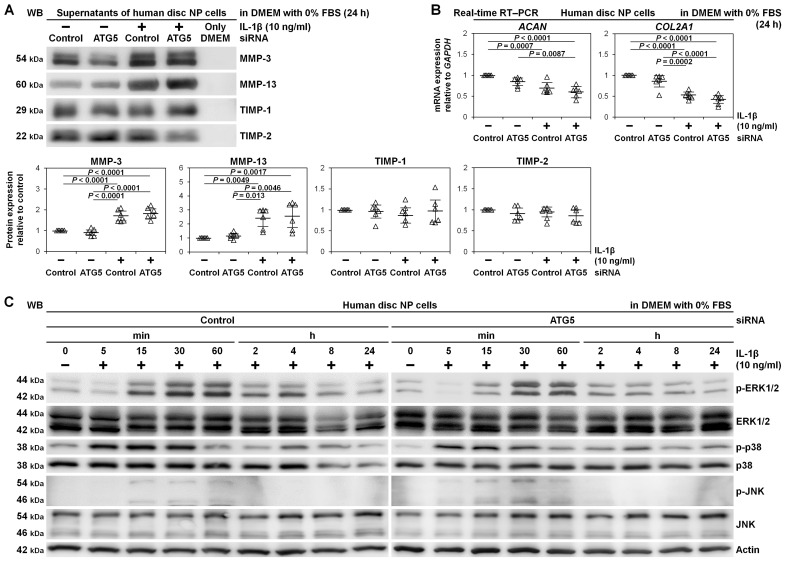
Unaffected matrix catabolism and MAPK-signaling pathways by autophagy inhibition through ATG5 knockdown in human disc NP cells. (**A**) Western blotting for catabolic MMP-3 and MMP-13 and anti-catabolic TIMP-1 and TIMP-2 in supernatant protein extracts from human disc NP cells after a 36 h transfection of ATG5 or non-targeting siRNA in 10% FBS-supplemented DMEM followed by culturing for 24 h in serum-free DMEM with or without 10 ng/mL IL-1β. Immunoblots shown are representative of experiments with similar results (*n* = 6). Changes in MMP-3, MMP-13, TIMP-1, and TIMP-2 protein expression of ATG5 RNAi and IL-1β treatment relative to non-targeting siRNA-transfected control without IL-1β are shown. Data are the mean ± 95% CI. One-way repeated-measures ANOVA and the Tukey-Kramer post-hoc test were used (*n* = 6). (**B**) Real-time RT–PCR for anabolic ACAN and COL2A1 in total RNA extracts from human disc NP cells after a 36 h transfection of ATG5 or non-targeting siRNA in 10% FBS-supplemented DMEM followed by culturing for 24 h in serum-free DMEM with or without 10 ng/mL IL-1β. GAPDH was used as an endogenous control. Changes in ACAN and COL2A1 (normalized to GAPDH) mRNA expression of ATG5 RNAi and IL-1β treatment relative to non-targeting siRNA-transfected control are shown. Data are the mean ± 95% CI. One-way repeated-measures ANOVA and the Tukey-Kramer post-hoc test were used (*n* = 6). (**C**) Time-course Western blotting for MAPK signaling-related ERK1/2, phosphorylated ERK1/2, p38, phosphorylated p38, JNK, and phosphorylated JNK in total protein extracts from human disc NP cells after a 36 h transfection of ATG5 or non-targeting siRNA in 10% FBS-supplemented DMEM followed by culturing for up to 24 h in serum-free DMEM with 10 ng/mL IL-1β. Actin was used as a loading control. Immunoblots shown are representative of experiments with similar results (*n* = 6).

We assessed whether ATG5 RNAi affected matrix metabolism in human disc NP cells. Western blotting in culture supernatants demonstrated IL-1β-induced matrix catabolism showing drastically released catabolic MMP-3 and MMP-13 compared to relatively unchanged anti-catabolic TIMP-1 and TIMP-2. However, these MMP-3 and MMP-13 production was unaffected by ATG5 RNAi. Similarly, TIMP-1 and TIMP-2 did not show distinct trends, suggesting insensitive catabolism and anti-catabolism by ATG5 knockdown (Figure 4A).

Real-time reverse transcription-polymerase chain reaction (RT–PCR), IL-1β induced mRNA down-regulation of anabolic ACAN encoding aggrecan (*p* = 0.0007) and COL2A1 encoding collagen type II alpha 1 chain (*p* < 0.0001). However, ATG5 RNAi did not present any trends in ACAN and COL2A1 gene expression, supporting unaffectedness by ATG5 knockdown (Figure 4B).

Since MAPKs are a key down-stream IL-1β effector [7], essential for MMP activation [43], we assessed whether ATG5-dependent autophagy did not truly involve matrix metabolism in human disc NP cells. In time-course Western blotting, IL-1β rapidly stimulated phosphorylation of extracellular signal-regulated kinase 1/2 (ERK1/2), p38, and c-Jun N-terminal kinase (JNK). Activations were maximum in p38 between 5 and 30 min and in ERK1/2 and JNK between 15 and 60 min and decreased thereafter, returning to basal levels by 24 h. However, there were no marked differences between the control and ATG5 RNAi groups, indicating no relevant MAPK-pathway involvement in ATG5-dependent autophagy (Figure 4C).

### 2.4. Induced Apoptosis and Senescence rather than Matrix Catabolism by Autophagy Inhibition through Chloroquine Supplementation in Human Disc NP Cells

**Figure 5:**
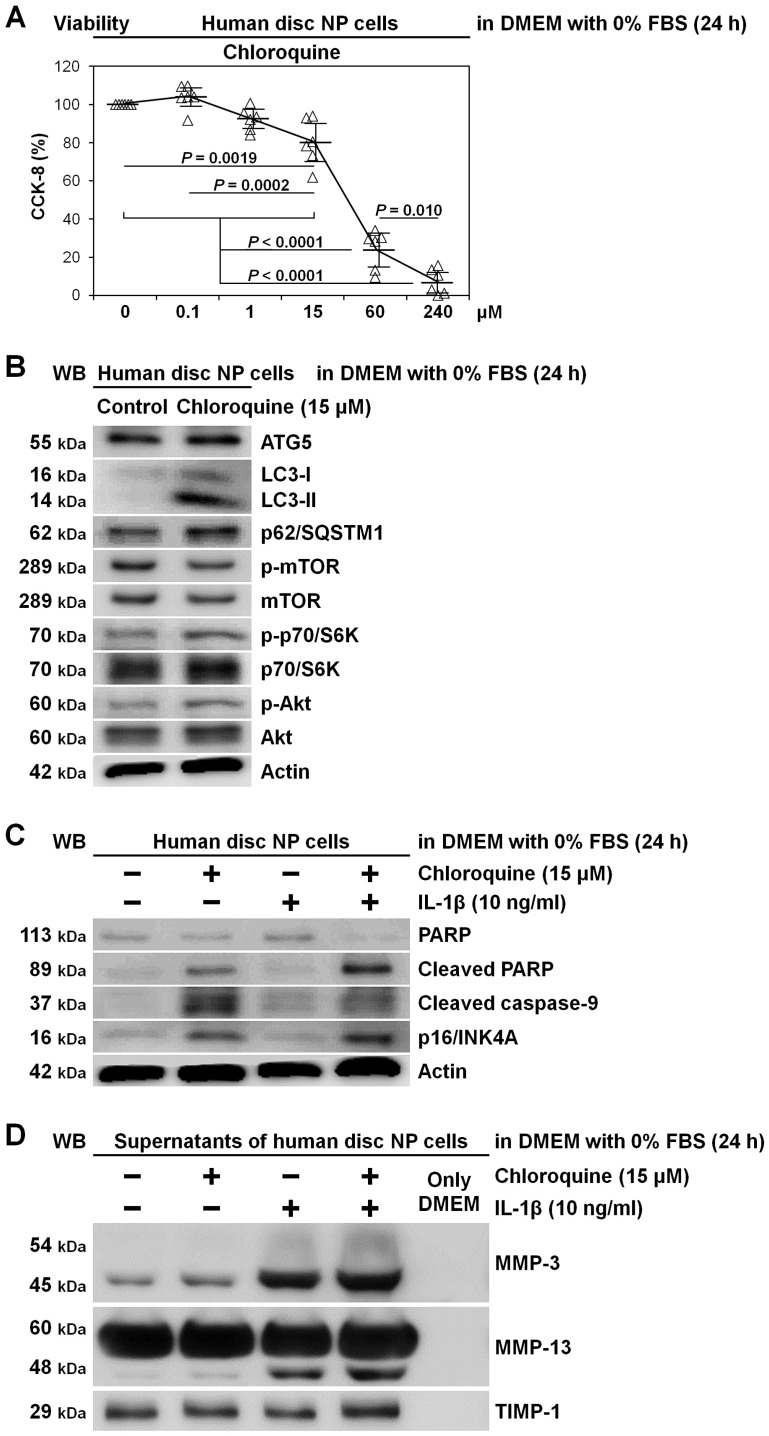
Induced apoptosis and senescence rather than matrix catabolism by autophagy inhibition through chloroquine supplementation in human disc NP cells. (**A**) Cell viability of human disc NP cells using CCK-8 after treatment of 0–240 μM chloroquine in serum-free DMEM with 0% FBS for 24 h. Changes in CCK-8 dehydrogenase activity of chloroquine treatment relative to the vehicle control are shown. Data are the mean ± 95% CI. One-way repeated-measures ANOVA and the Tukey–Kramer post-hoc test were used (*n* = 6). (**B**) Western blotting for autophagic ATG5, LC3, and p62/SQSTM1 and mTOR signaling-related mTOR, phosphorylated mTOR, p70/S6K, phosphorylated p70/S6K, Akt, and phosphorylated Akt in total protein extracts from human disc NP cells after culturing for 24 h in serum-free DMEM with or without 15 μM chloroquine. Actin was used as a loading control. Immunoblots shown are representative of experiments with similar results (*n* = 6). (**C**) Western blotting for pro-apoptotic cleaved PARP and cleaved caspase-9, anti-apoptotic PARP, pro-senescent p16/INK4A, and loading control actin in total protein extracts from human disc NP cells after culturing for 24 h in serum-free DMEM with or without 15 μM chloroquine. Immunoblots shown are representative of experiments with similar results (*n* = 6). (**D**) Western blotting for catabolic MMP-3 and MMP-13 and anti-catabolic TIMP-1 in supernatant protein extracts from human disc NP cells after 24 h culture in serum-free DMEM with or without 15 μM chloroquine. Immunoblots shown are representative of experiments with similar results (*n* = 6).

To support findings of early-stage autophagy inhibition by ATG5 RNAi in a different manner and approach, we tested lysosomotropic chloroquine for late-stage autophagy inhibition in human disc NP cells. In 0% FBS-supplemented DMEM requiring autophagy, chloroquine showed dose-dependent decreases in CCK-8-based cell viability, suspecting drug toxicity and autophagy inhibition. We thus selected 15 μM chloroquine as an effective but less toxic concentration (80.2% ± 11.1%, *p* = 0.0019) (Figure 5A). Chloroquine clearly displayed late-stage autophagy inhibition with increased LC3-II and p62/SQSTM1 but unchanged ATG5 expression and mTOR, p70/S6K, and Akt phosphorylation (Figure 5B). Under IL-1β stimulation, while apoptosis (cleaved PARP and caspase-9) and senescence (p16/INK4A) induction by chloroquine was distinct (Figure 5C), modified MMP and TIMP-1 release was unclear (Figure 5D).

### 2.5. Modified Autophagy-Related Protein Expression Based on the Patients’ Age in Human Disc NP Tissues

**Figure 6:**
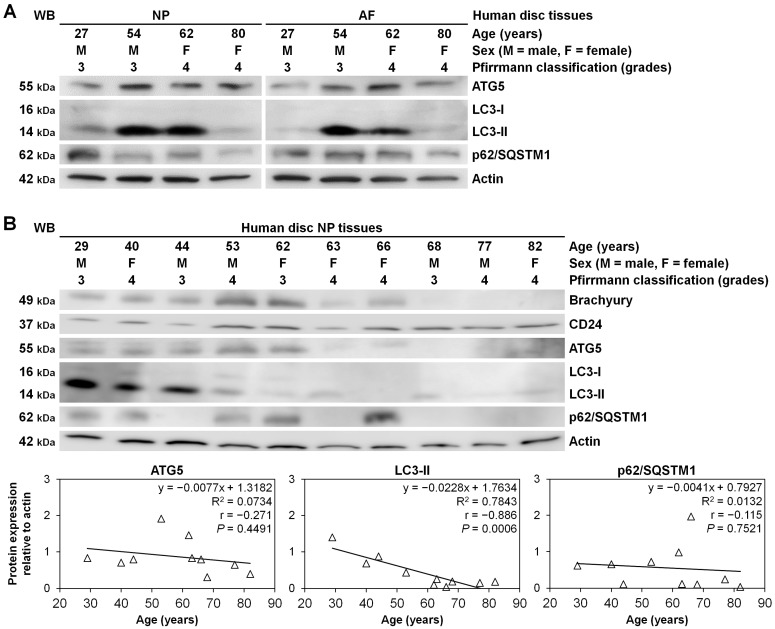
Modified autophagy-related protein expression based on the patients’ age in human disc NP tissues. (**A**) Western blotting for autophagic ATG5, LC3, and p62/SQSTM1 in total protein extract from human disc NP and AF tissues of patients who underwent lumbar spine surgery for degenerative disease. Actin was used as a loading control. Immunoblots show samples randomly selected (*n* = 4). (**B**) Western blotting for disc NP-phenotypic brachyury and CD24, autophagic ATG5, LC3, and p62/SQSTM1, and loading control actin in total protein extracts from human disc NP tissues. Immunoblots show samples randomly selected (*n* = 10). Protein expression levels of ATG5, LC3-II, and p62/SQSTM1 (normalized to actin) based on the patients’ age are shown. Regression analysis was used (*n* = 10).

We additionally examined autophagy involvement in human lumbar disc surgical specimens. In a limited number of patients, disc NP and AF tissues were simultaneously available, showing autophagy-related ATG5, LC3-II, and p62/SQSTM1 protein expression, levels of which were relatively constant between NP and AF samples in tested four cases in ages of 27–80 years (Figure 6A).

Then, in 10 cases of grade-3 and grade-4 disc NP-tissue surgical specimens in ages of 29–82 years, autophagic ATG5, LC3-II, and p62/SQSTM1 and disc NP-phenotypic brachyury and CD24 expression were all detected. Regression analysis demonstrated age-related decreases in LC3-II (*p* = 0.0006), suggesting impaired autophagy at least in older-aged discs (Figure 6B).

## 3. Discussion

This is the first loss-of-function study to examine specific roles of human disc NP cellular autophagy using RNAi and pharmacological modulation. Basal and serum starvation and inflammation-induced autophagy involvement in human disc NP cells were confirmed. Autophagy inhibition by ATG5 RNAi reduced disc NP-cell viability under stress conditions, requiring autophagy for nutrients and energy through apoptosis and senescence. However, ATG5-dependent autophagy inhibition did not modulate IL-1β-induced disc NP-cell catabolic MMP production, anabolic ACAN and COL2A1 mRNA down-regulation, and MAPK-pathway activation. Late-stage autophagy inhibition by chloroquine presented similar findings of early-stage autophagy inhibition by ATG5 knockdown. Age-related decreases in autophagic LC3-II protein were identified in human disc NP and AF tissues. The current study provides information to develop future biological autophagy-modulating therapies for degenerative disc disease.

In this study, RNAi-mediated ATG5 knockdown, excluding off-target effects from consistent findings of different siRNA sequences, inhibited human disc cellular autophagy without mTOR-signaling modulation. ATG5 is essential for autophagic vesicle formation [19]. Knockout of ATG5 results in total autophagy inhibition [44]. Thus, ATG5 is a common target in autophagy gene-editing studies [45]. While ATG5 is involved in immune responses and apoptosis [45], little evidence regarding the interaction between ATG5 and mTOR exists. However, this is the subject studied in the future.

Under serum deprivation and inflammation, ATG5 knockdown-induced autophagy inhibition reduced human disc-cell viability. Although the discrepancy between in vitro cell-growth culture and in vivo avascular, low-nutrient environment is often cautioned [31], autophagy requirements for disc-cell survival in experimental nutrition-limited, inflammatory conditions have been confirmed. Then, ATG5 knockdown-induced decreases in disc-cell viability involved apoptosis and senescence. This apoptosis was driven through the mitochondrial pathway, which is consistent with human chondrocyte autophagy inhibition [26]. Anti-apoptotic and anti-senescent effects of autophagy are known in many cell types [16,17,19,24,25,26]. Human degenerative disc cellular protection through ATG5-dependent autophagy further justifies autophagy-modulating therapies.

We previously reported human disc cellular anti-apoptosis and anti-senescence with Akt and autophagy induction by mTORC1 inhibition [24], which was primarily Akt-dependent [25]. Integrated with this study findings, mTORC1 inhibition-induced cellular protection would result from collaborative effects of Akt and autophagy. Suppressed mTORC1 triggers autophagy, also exerting translation-stimulating p70/S6K deactivation [20]. Indeed, mTOR-hypomorphic mice at approximately 25% expression exhibit increased lifespan and decreased organ senescence biomarkers, including p16/INK4A [46]. The p70/S6K deletion also extends lifespan [47]. Then, through the negative feedback loop between p70/S6K and the class-I PI3K, mTORC1 inhibition-mediated Akt induction stimulates cell survival by blocking pro-apoptotic BCL2-associated death promoter protein and through effects on transcription factors, forkhead box O and p53 [21]. The Akt also enhances cell proliferation by inhibiting negative cell-cycle regulators, p27/KIP1 and p21/WAF1/CIP1 [21]. All these findings support selective mTORC1-inhibition therapies, leading to autophagy induction, as a key strategy to protect human disc cells.

Meanwhile, unaffected matrix catabolism by ATG5 knockdown is controversial. We found non-significant changes in human disc-cell ACAN and COL2A1 gene expression by ATG5 RNAi, whereas Sasaki H et al. reported ATG5 RNAi-mediated significant ACAN and COL2A1 down-regulation and reduced aggrecan and collagen type II protein expression in human chondrocytes [26]. Although we measured released catabolic MMP-3 and MMP-13 and anti-catabolic TIMP-1 and TIMP-2 protein amounts, all were unchanged by ATG5 RNAi. We further monitored MAPK-signaling pathways, major MMP production, and activation cascades [7,43]; nevertheless, ERK1/2, p38, and JNK phosphorylation levels were all insensitive to ATG5 RNAi. Consequently, human disc-cell matrix metabolism would not involve ATG5-dependent autophagy. However, this could depend on the cell type, requiring further investigation.

Only a few reports have described autophagy and mTOR signaling-mediated matrix metabolism [18,24,25]. Our previous study demonstrated disc cellular mTORC1 inhibition-mediated reduction in MMP expression and activation [24]. This MMP suppression could be explained by decreased p70/S6K-dependent translation [20] and increased Akt-dependent MAPK-pathway inactivation [21]. Based on the current study, mTORC1 suppression-induced anti-matrix catabolism would result from modified PI3K/Akt/mTOR signaling rather than autophagy in human disc cells.

Autophagy-inhibition findings by early-stage ATG5-RNAi and late-stage chloroquine supplementation were relatively consistent. However, chloroquine-induced modest increases in catabolic MMPs may come from disc-cell toxicity. Chloroquine does not inhibit autophagy specifically but lysosomal function broadly [19]. Nevertheless, this study provides interesting information on anti-malarial, anti-rheumatic, and anti-viral chloroquine [48] on intervertebral disc disease and low back pain.

Our human disc-tissue Western blotting detected clinically relevant autophagy. Despite the increased interest [16,17,19], actual autophagy involvement in disc degeneration is unknown [18]. Autophagy levels, primarily determined by LC3-II, showed age-related decreases in human grade-3 and grade-4 disc specimens. In older-aged discs, cells might lose the potential of stress-response autophagy. These findings suggest the importance of age in patient selection for autophagy-modulating therapies against degenerative disc disease.

A limitation of this study was it lacked in vivo data. Mice having knockout of Atg5 in disc cells would be helpful to sophisticate our results. In addition, animal model studies of disc degeneration, including in vivo models [8,9,10,13,49] and ex vivo models [50,51], would further reveal the impact of disc cellular autophagy. Another limitation is monolayer cell culture. Three-dimensional culture systems are favorable to simulate the physiological disc environment, although it is difficult to analyze molecular signaling using alginate gel beads. An additional limitation was the relatively small sample size with limited consideration of variations in age, sex, and disc degeneration grade, warranting a more careful interpretation. However, the present findings were consistent regardless of these issues.

## 4. Materials and Methods

### 4.1. Ethics Statement

All experimental procedures were performed under the approval and guidance of the Institutional Review Board (160004, 16 May 2016) at Kobe University Graduate School of Medicine. Written informed consent was obtained from each patient in accordance with the principles of the Declaration of Helsinki and the laws and regulations of Japan.

### 4.2. Antibodies and Reagents

The antibodies and reagents used are listed in Table S1.

### 4.3. Cells

Human disc NP cells were obtained from specimens of patients who underwent lumbar interbody fusion surgery for degenerative disease (*n* = 25: age, 63.0 ± 5.3 (range, 27–80) years; 8 males and 17 females; Pfirrmann degeneration grade [30], 3.4 ± 0.2 (range, 3–4)). Immediately after surgery, human disc NP tissues were carefully collected from discarded surgical waste and digested in 1% penicillin/streptomycin-supplemented DMEM with 10% FBS and 0.114% collagenase type 2 for 1 h at 37 °C. Isolated first-passage cells were grown to ~80% confluence as a monolayer in 1% penicillin/streptomycin-supplemented DMEM with 10% FBS at 37 °C under 2% O_2_ and used for evaluation following pre-culturing for 72 h (60 h in DMEM with 10% FBS, followed by 12 h with 1% FBS).

Cells were grown to 5.0 × 10^3^/well (96-well plate) for viability analysis, 1.5 × 10^5^/well (6-well plate) for protein extraction and RNA isolation, and 1.2 × 10^4^/well (8-well chamber) for staining, and were distributed randomly. In each experiment, six-cell samples from 6 different patients were used throughout the treatment (*n* = 6). Cell samples collected abundantly were used across experiments. Consequently, a total of 25 patient samples were used for the in vitro experiments.

To simulate clinically relevant disease conditions of cellular stress [33], serum deprivation and/or pro-inflammatory IL-1β supplementation were applied.

As autophagy-inhibiting treatments, RNAi of ATG5 and lysosomotropic chloroquine supplementation were tested. Chloroquine was soluble in water.

After 36 h RNAi or 24 h drug treatment, cells were applied to Western blotting for autophagy and mTOR signaling and viability assay using CCK-8. After additional 24 h serum starvation and/or IL-1β stimulation, cells were applied to CCK-8 for viability, Western blotting for apoptosis and senescence in total protein extracts and matrix catabolism in supernatant protein extracts, TUNEL staining for apoptosis, SA-β-gal staining for senescence, and real-time RT–PCR for matrix components.

### 4.4. Tissues

Portions of human disc NP and AF tissues surgically obtained from the lumbar spine were carefully dissected and directly used for protein extraction (*n* = 14: age, 57.6 ± 9.2 (range, 27–82) years; 7 males and 7 females; Pfirrmann degeneration grade [30], 3.6 ± 0.3 (range, 3–4)).

### 4.5. RNAi

Cells were applied to RNAi using two siRNAs with different sequences against autophagy-essential ATG5. Non-targeting siRNA was used as a negative control. The siRNA sequences are listed in Table S2. As the reverse transfection technique for effective siRNA delivery, cells suspended in DMEM with 10% FBS was added to Opti-minimal essential medium I with Lipofectamine RNAiMAX and the siRNA, cultured for 36 h, and used for analysis. Applied siRNA amounts were 60 (6-well plate) and 2 (96-well plate) pmol/well.

### 4.6. Cell Viability Assay

Cell viability was assessed by CCK-8 dehydrogenase activity, the absorbance of which (450 nm) was measured using the Model 680 microplate reader.

### 4.7. Protein Extraction

Cells were scraped off on ice in 3-(N-morpholino)propanesulfonic acid buffer containing protease and phosphatase inhibitors. Soluble proteins were collected after 15 min 20,000× *g* centrifugation at 4 °C. Non-serum-containing culture media were also collected after 10 min 1000× *g* centrifugation at 4 °C to remove cellular debris and concentrated using Amicon Ultra spin columns.

Tissues were homogenized using the MS-100R bead-beating disrupter for 30 s twice at 4 °C in the T-PER tissue protein extraction reagent with protease and phosphatase inhibitors. Soluble proteins were collected after 15 min 20,000× *g* centrifugation at 4°C.

Protein concentration was determined by the bicinchoninic acid assay. Samples were stored at −80 °C.

### 4.8. Sodium Dodecyl Sulfate (SDS)–Polyacrylamide Gel Electrophoresis and Western Blotting

Equal 30-µg amounts of protein were mixed with the electrophoresis sample buffer and boiled for 5 min before loading onto a 7.5–15.0% polyacrylamide gel. Separated proteins in the Tris–glycine–SDS buffer system were transblotted to a polyvinylidene difluoride membrane electrically and probed with primary antibodies for 12 h in 4 °C (1:200–1:1000 dilution) followed by secondary antibodies (1:2000 dilution). Signals were visualized by enhanced chemiluminescence. Images were obtained using the Chemilumino analyzer LAS-3000 mini. Band intensity was quantified using the ImageJ software (http://rsbweb.nih.gov/ij/, 16 May 2016).

Western blotting was designed to analyze intracellular expression of disc NP notochord-related brachyury and CD24 [32], autophagy-related ATG5, LC3, and p62/SQSTM1 [19], mTOR signaling-related mTOR, phosphorylated mTOR, p70/S6K, phosphorylated p70/S6K, Akt, and phosphorylated Akt [20], apoptosis-related PARP [37], cleaved PARP, cleaved caspase-9 [38], BCL2 [39], and BAX [39], senescence-related p16/INK4A [40], p21/WAF1/CIP1 [41], and p53 [42], MAPK-related ERK1/2, phosphorylated ERK1/2, p38, phosphorylated p38, JNK, and phosphorylated JNK in total cell or tissue protein extracts [7,43]. Western blotting was also designed to analyze released expression in supernatant protein extracts of catabolic MMP-3 and MMP-13 and anti-catabolic TIMP-1 and TIMP-2 [7].

### 4.9. TUNEL Staining

Cells fixed with 10 min 4% paraformaldehyde were used for fluorescein-labeled TUNEL staining with 4′,6-diamidino-2-phenylindole (DAPI) counterstaining for apoptotic fragmented DNA detection [35]. Images were photographed by the BZ-X700 microscope. The percentage of TUNEL-positive cells was calculated relative to the number of DAPI-positive total cells. Both countings were performed in six random low-power fields (×100) using ImageJ.

### 4.10. SA-β-Gal Staining

Cells after fixation were assigned to cytochemical SA-β-gal staining at pH 6 for replicative senescence identification [36]. The percentage of SA-β-gal-positive cells was similarly calculated in six random low-power fields (×100).

### 4.11. RNA Isolation and Real-Time RT–PCR

Total RNA was extracted using the RNeasy mini kit, and 0.1 µg of RNA was reverse-transcribed with random primers. Messenger RNA expression levels of anabolic ACAN and COL2A1 relative to glyceraldehyde 3-phosphate dehydrogenase (GAPDH) were assessed by real-time RT–PCR using SYBR^TM^ Green fluorescent dye. Good feasibility of GAPDH as an endogenous control for disc cells was established previously [49]. The primer sequences were obtained from prior reports [24,25], as listed in Table S2. Measurements were performed using the ABI Prism 7500 real-time PCR system. Melting curve analysis was performed using the Dissociation Curves software to ensure the amplification of only a single product. Relative mRNA expression was analyzed using the 2−∆∆Ct method [52]. The non-targeting siRNA-transfected control sample value was set as 1.

### 4.12. Statistical Analysis

In vitro, cells were analyzed in duplicate. Single data value was obtained by averaging these 2 technical replicates. Experiments were conducted six times using 6 different patient samples (*n* = 6). In Western blotting and staining, immunoblots and images shown are representative of six similar results from these 6 biological replicates. In vivo, Western blotting was performed using 14 different patient samples (*n* = 14).

In vitro, CCK-8 cell viability, Western blotting protein expression, and real-time RT–PCR gene expression were calculated as relative values of the control while staining positivity was compared using replicates from the same donors; therefore, paired *t*-test (comparison between 2 groups) or one-way repeated measures analysis of variance (ANOVA) and the Tukey–Kramer post-hoc test (comparison between ≥3 groups) were used. In vivo, Western blotting for protein expression based on the patients’ age was evaluated by regression analysis.

Data are expressed as the mean ± 95% confidence interval (CI). The *p*-values < 0.05 were regarded as statistically significant using IBM SPSS Statistics 23.0 (IBM, Armonk, NY, USA). 

## 5. Conclusions

The primary role of human disc cellular autophagy is to maintain homeostasis through anti-apoptosis and anti-senescence rather than to regulate matrix metabolism through MAPK-signaling pathways. Autophagic cellular protection is a potent molecular treatment for degenerative disc disease.

## Data Availability

The data presented in this study are available on reasonable request from the corresponding author.

## Supplementary Materials

The following are available online at https://www.mdpi.com/article/10.3390/ijms22083965/s1, Table S1: List of antibodies, reagents, and instruments used. Table S2: List of small interfering RNA (siRNA) and real-time reverse transcription (RT)–polymerase chain reaction (PCR) primer sequences used.
