# Supplementary material for: Inhibition of Autophagy at Different Stages by ATG5 Knockdown and Chloroquine Supplementation Enhances Consistent Human Disc Cellular Apoptosis and Senescence Induction rather than Extracellular Matrix Catabolism
Source: Int J Mol Sci. 2021 Apr 12;22(8):3965. doi: 10.3390/ijms22083965 (PMC8069032; doi:10.3390/ijms22083965)
Supplement: Supplementary file 1 [file ijms-22-03965-s001.zip › Supplemental Tables.pdf]

Supplemental Table 1. List of antibodies, reagents, and instruments used.

Product	Catalog number	Manufacturer
Antibody		
ATG5 (rabbit, monoclonal)	12994	Cell Signaling Technology (Danvers, MA)
LC3 (rabbit, monoclonal)	3868	Cell Signaling Technology (Danvers, MA)
mTOR (rabbit, monoclonal)	2983	Cell Signaling Technology (Danvers, MA)
Phosphorylated mTOR at Ser2448 (rabbit, polyclonal)	2971	Cell Signaling Technology (Danvers, MA)
p70/S6K (rabbit, monoclonal)	2708	Cell Signaling Technology (Danvers, MA)
Phosphorylated p70/S6K at Thr389 (rabbit, monoclonal)	9234	Cell Signaling Technology (Danvers, MA)
Akt (rabbit, monoclonal)	4691	Cell Signaling Technology (Danvers, MA)
Phosphorylated Akt at Ser473 (rabbit, monoclonal)	4060	Cell Signaling Technology (Danvers, MA)
PARP (rabbit, monoclonal)	9532	Cell Signaling Technology (Danvers, MA)
Cleaved PARP (rabbit, monoclonal)	5625	Cell Signaling Technology (Danvers, MA)
Cleaved caspase-9 (rabbit, polyclonal)	9501	Cell Signaling Technology (Danvers, MA)
BCL2 (rabbit, polyclonal)	2872	Cell Signaling Technology (Danvers, MA)
BAX (rabbit, polyclonal)	2774	Cell Signaling Technology (Danvers, MA)
p21/WAF1/CIP1 (rabbit, monoclonal)	2947	Cell Signaling Technology (Danvers, MA)
p53 (mouse, monoclonal)	2524	Cell Signaling Technology (Danvers, MA)
TIMP-2 (rabbit, monoclonal)	5738	Cell Signaling Technology (Danvers, MA)
ERK1/2 (rabbit, monoclonal)	4695	Cell Signaling Technology (Danvers, MA)
Phosphorylated ERK1/2 at Thr202/Tyr204 (rabbit, monoclonal)	4370	Cell Signaling Technology (Danvers, MA)
p38 (rabbit, monoclonal)	8690	Cell Signaling Technology (Danvers, MA)
Phosphorylated p38 at Thr180/Tyr182 (rabbit, monoclonal)	4511	Cell Signaling Technology (Danvers, MA)
JNK (rabbit, polyclonal)	9252	Cell Signaling Technology (Danvers, MA)
Phosphorylated JNK at Thr183/Tyr185 (rabbit, monoclonal)	4668	Cell Signaling Technology (Danvers, MA)
p62/SQSTM1 (mouse, monoclonal)	ab56416	Abcam (Cambridge, UK)
MMP-3 (rabbit, monoclonal)	ab52915	Abcam (Cambridge, UK)
MMP-13 (rabbit, polyclonal)	ab39012	Abcam (Cambridge, UK)
Brachyury (goat, polyclonal)	sc-17743	Santa Cruz Biotechnology (Santa Cruz, CA)
CD24 (rabbit, polyclonal)	sc-11406	Santa Cruz Biotechnology (Santa Cruz, CA)
p16/INK4A (mouse, monoclonal)	sc-1661	Santa Cruz Biotechnology (Santa Cruz, CA)
TIMP-1 (rabbit, polyclonal)	sc-5538	Santa Cruz Biotechnology (Santa Cruz, CA)
β-Actin (mouse, monoclonal)	A5441	Sigma-Aldrich (St. Louis, MO)

Cell culture		
DMEM	D5796	Sigma-Aldrich (St. Louis, MO)
FBS	F2442	Sigma-Aldrich (St. Louis, MO)
Chloroquine diphosphate salt	C6628	Sigma-Aldrich (St. Louis, MO)
Penicillin/streptomycin	26253-84	Nacalai Tesque (Kyoto, Japan)
Collagenase type 2	LS004176	Worthington Biochemical (Lakewood, NJ)
Human recombinant IL-1β	ALX-520-001	Enzo Life Science (Farmingdale, NY)
CCK-8	CK04	Dojindo Laboratories (Kumamoto, Japan)
Model 680 microplate reader		Bio-Rad (Hercules, CA)
RNAi		
Lipofectamine RNAiMAX transfection reagent	13778150	Thermo Fisher Scientific (Waltham, MA)
Opti-minimal essential medium I	3198070	Thermo Fisher Scientific (Waltham, MA)
Stealth RNAi siRNA negative control	12935	Thermo Fisher Scientific (Waltham, MA)
Stealth RNAi siRNAs		Thermo Fisher Scientific (Waltham, MA)
Western blotting		
3-(*N*-Morpholino)propanesulfonic acid	23438-64	Nacalai Tesque (Kyoto, Japan)
Protease inhibitor cocktail	25955-11	Nacalai Tesque (Kyoto, Japan)
Tris(hydroxymethyl)aminomethane	35434-21	Nacalai Tesque (Kyoto, Japan)
Chemi-Lumi One Super	02230-30	Nacalai Tesque (Kyoto, Japan)
Phosphatase inhibitor cocktails 2	P5726	Sigma-Aldrich (St. Louis, MO)
Phosphatase inhibitor cocktails 3	P0044	Sigma-Aldrich (St. Louis, MO)
Glycine	12-1210	Sigma-Aldrich (St. Louis, MO)
T-PER tissue protein extraction reagent	78510	Thermo Fisher Scientific (Waltham, MA)
Pierce BCA protein assay kit	23227	Thermo Fisher Scientific (Waltham, MA)
Polyacrylamide gel	SDG-581	Bio Craft (Tokyo, Japan)
4× Laemmli sample buffer	1610747	Bio-Rad (Hercules, CA)
SDS	191-07145	Wako (Osaka, Japan)
Polyvinylidene difluoride membrane	10600021	GE Healthcare (Chicago, IL)
Anti-rabbit secondary antibody	NA934	GE Healthcare (Chicago, IL)
Anti-mouse secondary antibody	NA931	GE Healthcare (Chicago, IL)
Anti-goat secondary antibody	sc-2020	Santa Cruz Biotechnology (Santa Cruz, CA)
Amicon Ultra spin columns	UFC200324	Merck (Darmstadt, Germany)
MS-100R bead-beating disrupter		Tomy Seiko (Tokyo, Japan)
LAS-3000 mini		Fujifilm (Tokyo, Japan)
Staining		
In situ cell death detection kit, fluorescein	11684795910	Roche (Basel, Switzerland)
DAPI	11034-56	Nacalai Tesque (Kyoto, Japan)
SA-β-gal staining kit	9860	Cell Signaling Technology (Danvers, MA)
4% Paraformaldehyde phosphate buffer solution	163-20145	Wako (Osaka, Japan)
BZ-X700 microscope		Keyence (Osaka, Japan)

Real-time RT–PCR		
RNeasy mini kit	74104	Qiagen (Hilden, Germany)
High-capacity cDNA reverse transcription kit	4368813	Thermo Fisher Scientific (Waltham, MA)
Power SYBR Green master mix	4367659	Thermo Fisher Scientific (Waltham, MA)
Custom DNA primers		Thermo Fisher Scientific (Waltham, MA)
		Takara Bio (Shiga, Japan)
Applied Biosystems 7500 real-time PCR system		Thermo Fisher Scientific (Waltham, MA)

ATG5 = autophagy-related gene 5; BAX = BCL2-associated X protein; BCA = bicinchoninic acid; BCL2 = B-cell lymphoma 2; CCK-8 = cell counting kit-8; cDNA = complementary DNA; DAPI = 4′,6-diamidino-2-phenylindole; DMEM = Dulbecco's modified Eagle's medium; ERK = extracellular signal-regulated kinase; FBS = fetal bovine serum; IL-1β = interleukin-1 beta; JNK = c-Jun N-terminal kinase; LC3 = light chain 3; MMP = matrix metalloproteinase; mTOR = mammalian target of rapamycin; p62/SQSTM1 = p62/sequestosome 1; p70/S6K = p70/ribosomal S6 kinase; PARP = poly (ADP-ribose) polymerase; RNAi = RNA interference; RT–PCR = reverse transcription–polymerase chain reaction; SA-β-gal = senescence-associated beta-galactosidase; SDS = sodium dodecyl sulfate; siRNA = small interfering RNA; TIMP = tissue inhibitor of metalloproteinases.

Supplemental Table 2. List of small interfering RNA (siRNA) and real-time reverse transcription (RT)–polymerase chain reaction (PCR) primer sequences used.

Gene			Sequence (5' to 3')
siRNA			
ATG5	No. 1	Sense	GGUUUGGACGAAUUCCAACUUGUUU
		Anti-sense	AAACAAGUUGGAAUUCGUCCAAACC
	No. 2	Sense	GAUCACAAGCAACUCUGGAUGGGAU
		Anti-sense	AUCCCAUCCAGAGUUGCUUGUGAUC
Real-time RT–PCR primer			
ACAN		Forward	AAGAATCAAGTGGAGCCGTGTGTC
		Reverse	TGAGACCTTGTCCTGATAGGCACT
COL2A1		Forward	AAGGTGCTTCTGGTCCTGCTG
		Reverse	GGGATTCCATTAGCACCATCTTTG
GAPDH		Forward	GAGGCCGGTGCTGAGTAT
		Reverse	GCGGAGATGATGACCCTTTTGG

ACAN = aggrecan; ATG5 = autophagy-related gene 5; COL2A1 = collagen type II alpha 1 chain; GAPDH = glyceraldehyde 3-phosphate dehydrogenase.
